# Foreign Bodies in the Eye

**Published:** 1889-10-19

**Authors:** 


### FOREIGN BODIES IN THE EYE.

Mr. Simeon Snell writes a timely letter to the *Lancet* (October 5, 1889) "On the Use of the Electro-Magnet in Ophthalmic Surgery," especially as Mr. Snell observes that he sees no mention of this plan in a late clinical lecture on the removal of foreign bodies from the eye. Mr. Snell says that from different regions of the eye he has removed by the magnet seventy-three fragments of steel or iron, of which number eight have been from the iris and anterior chamber. We quite agree with the writer that the use of the magnet is invaluable, and that by its use fragments of metal may be not only removed, but also discovered, which could not have been removed nor discovered in any other manner. So far as we are aware, most text-books on ophthalmic surgery afford space for a notice of the magnet; at least all text-books of recent date. But on referring to older authors, Tyrrell, "On Diseases of the Eye," for instance, a capital book in its day, we do not find any mention made of this excellent method of removing fragments of metal from the organ. It is a comparatively recent advance in ophthalmic surgery.

